# Supplementary material for: Prediction of Deep Myometrial Infiltration, Clinical Risk Category, Histological Type, and Lymphovascular Space Invasion in Women with Endometrial Cancer Based on Clinical and T2-Weighted MRI Radiomic Features
Source: Cancers (Basel). 2023 Apr 8;15(8):2209. doi: 10.3390/cancers15082209 (PMC10136642; doi:10.3390/cancers15082209)
Supplement: Supplementary file 1 [file cancers-15-02209-s001.zip › cancers-2277637-supplementary.pdf]

Supplementary material

Features for DMI classification

The fitcauto method shows randomness, several runs may be needed to achieve better results. Also, the number of features need to be predefined if univariate model selection method was employed. In this study, eight features were adopted, other number of features may achieve better results. For the LASSO method, because cross-validation was implemented, the results also show randomness. The results in this study were from a typical 10-fold cross-validation method.

In the DMI prediction analysis, the training dataset includes 292 cases. 199 of these cases were no deep invasion, and 99 cases with deep invasion (Table 1). Using the best selection method, 8 most important features were selected as shown in Figure S1, where the importance is ranked from left to right side. In this case, the most important feature was FOS_Imean_HLL, followed by GLSZM_SzoneHiGl_64gl, GLRLM_LRLGLE_LLL_16gl, FOS_CV_HLL, GLSZM_LzoneLogl_LLL_16gl, GLCM_sumAvg_4gl, GLCM_Contra_LLH_8gl, and GLRLM_SRHGLE_16gl. The following list shows the meaning of these features.

GLSZM represents: Gray Level Size Zone.

Contra: CONTRAST group

sumAvg: sum average

16gl: 16 grey level of the image, 4,8,32,64,128,256gl means the corresponding number of grey level.

SRHGLE: short run high grey level emphasis

GLRLM: Gray Level Run Length Matrix.

LRLGLE: long run low grey level emphasis

LzoneLogl: Large Zone Low Gray-level Emphasis

GLSZM: grey-level size-zone matrix

FOS: first order statistics;

Imean: image intensity mean value;

CV: coefficient of variation

GLCM: gray-level co-occurrence matrix

LzoneLogl: Large Zone Low Gray-level Emphasis;

SzoneHiGl: Small Zone High Gray-level Emphasis;

In addition, in the radiomic feature name, 'LLH' means that the lowpass (scaling) filter with down sampling is applied to the rows of the 3D image, followed by the low pass (wavelet) filter with down sampling applied to the columns of image. Finally, the high pass filter with down sampling is applied to the 3rd dimension of the image. It is the same for ‘LLL’ etc.

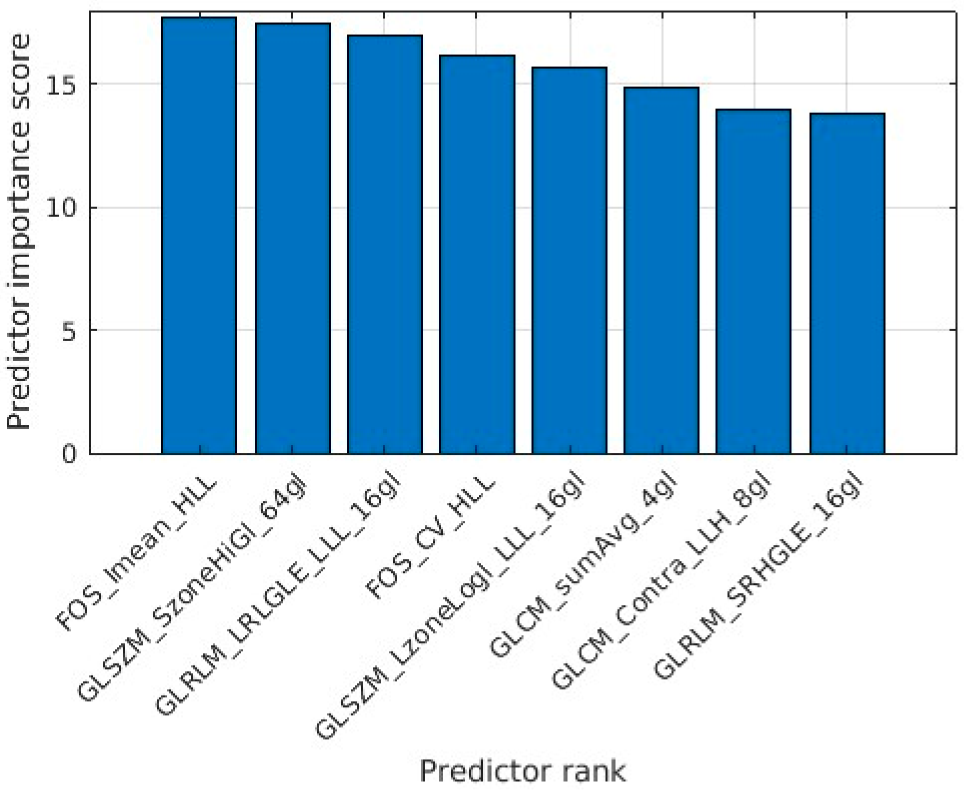

Figure S1. Feature importance bar plot for DMI prediction. Feature importance score for the most important 8 features for endometrial cancer patient DMI prediction using the univariate selection method. The Y axis is the importance score measured by the -log(p) value. A p-value of the test statistic indicates that the corresponding predictor variable is dependent on the response variable. The X axis is the name of the feature.

For the LASSO method to classify DMI, the maximum number of features was set to be 8. Seven radiomic features were selected in addition to intercept. There 7 features were FOS_Skew, GLCM_sumAvg_4gl, GLRLM_SRHGLE_16gl, GLSZM_LzoneLogl_LLL_16gl, FOS_Ener_LLH_8gl, FOS_Ener_LLH_16gl, and original_shape_Flatness. In these feature name, Ener denotes energy, and SRHGLE is short_run_high_grey_level_emphasis.

Features for risk classification

Using training dataset, as we classified the cases into two risk groups, i.e., low-risk and high-risk groups, 150 of 413 were low-risk cases. The testing study included 81 cases, of which 41 cases were low risk cases (Table 1). Before applying fitcauto method, the univariate selection method was applied and the most important 8 features were used as shown in Figure S2. The most important feature for the classification was cancer grade, following by:

GLCM_Homoge_LLH_16gl, GLSZM_LargeZone_LLH_128gl,NGTDM_Contra_HLH_8gl, GLCM_Dissim_HLH_8gl, GLCM_invVar_HLH_16gl, GLCM_Homoge_HLH_8gl, and FOS_Entr_HLH_8gl. The abbreviations in the names are listed in the following:

NGTDM: Neighbouring Gray Tone Difference Matrix;
Homoge: Homogeneity/Inverse Difference Moment;
Dissim: Dissimilarity;
Entr: entropy;
FOS: first order statistics;
GLCM: gray-level co-occurrence matrix;
LRE: long run emphasis;
GLRLM: gray level run length matrix;

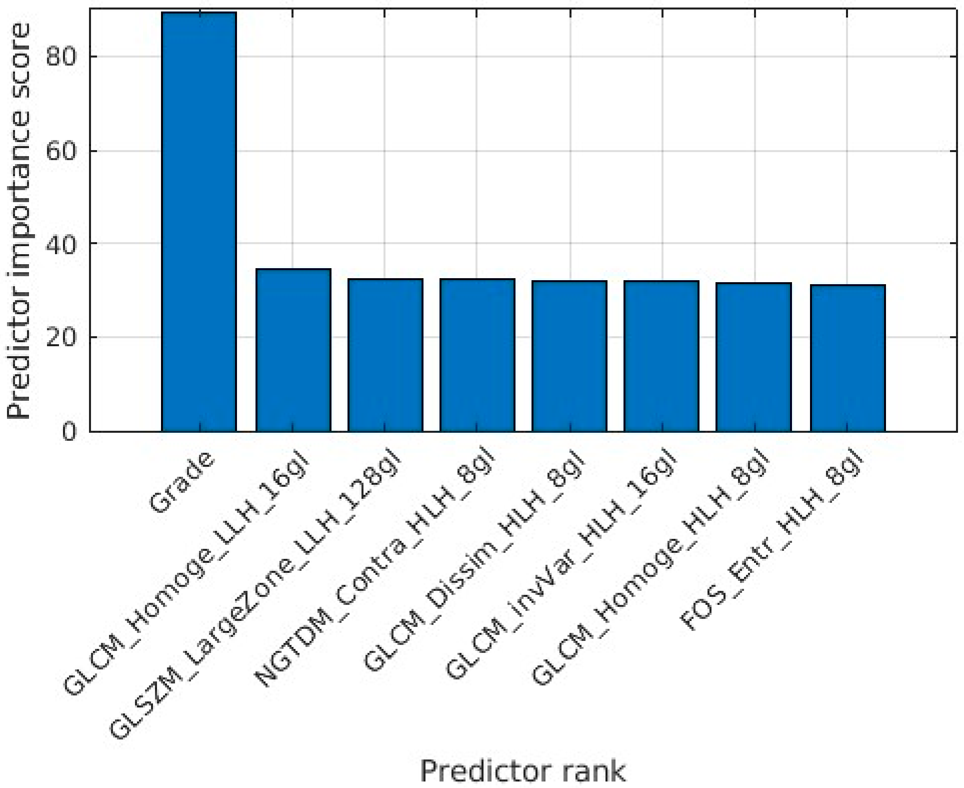

Figure S2. Feature importance bar plot for patient risk prediction. Feature importance score for the 8 most important features in the patient risk classification. The Y axis is the importance score measured by the -log(p) value. The X axis is the name of the feature.

For high-risk endometrial cancer patient classification, using LASSO method with 8 maximum number of features, the selected features were: GLCM_difEnt_LLH_8gl, FOS_Imean_HLL, FD_max_HLL_32gl, NGTDM_Contra_HLH_8gl, GLCM_Dissim_HLH_8gl, GLCM_invVar_HLH_16gl, original_shape_LeastAxisLength, and Age. In the these feature names, difEnt is difference Entropy; invVar is Inverse Variance, and FD is fractal dimension.

Feature for histological type classification

The purpose of the histological type study was to design a machine learning method to classify endometrioid type of cancer from the other type of endometrial cancer. For the training datasets, there were 301 endometrioid cases and 111 other types (Table 1). In the analysis, endometrioid type was assigned as 0, while the other cases were given as 1 in the target variable. The selected 8 features were displayed in Figure S3.

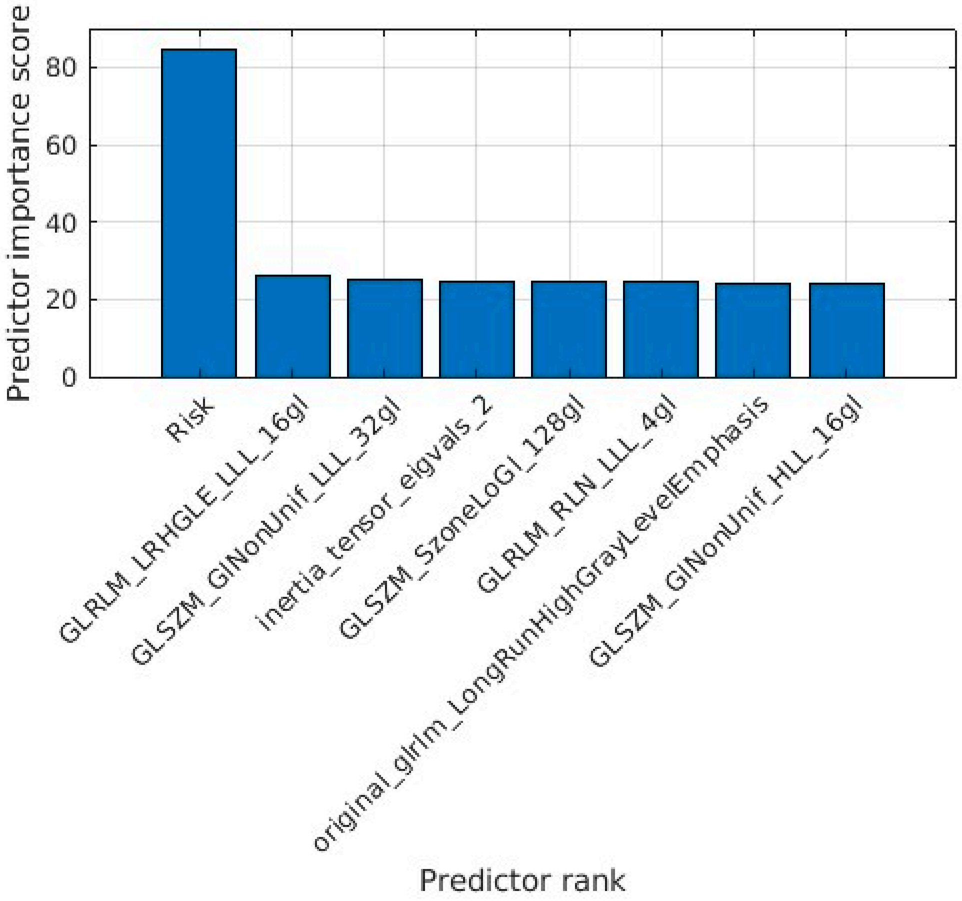

Figure S3. Feature importance bar plot for histological type prediction. Feature importance score for the most important 8 features for histological type classification. The Y axis is the importance score measured by the -log(p) value. The X axis is the name of the feature. Without including cancer grade.

Using the univariate selection method, the 8 selected features for the fitcauto method were: Risk, GLRLM_LRHGLE_LLL_16gl, GLSZM_GlNonUnif_LLL_32gl, inertia_tensor_eigvals_2, GLSZM_SzoneLoGl_128gl, GLRLM_RLN_LLL_4gl, original_glrlm_LongRunHighGrayLevelEmphasis, and GLSZM_GlNonUnif_HLL_16gl. In these feature names, GINonUnif is Grey Level non-uniformity, GLRLM denotes gray level run length matrix, and LRHGLE is the long run high grey level emphasis.

For LASSO method, 8 selected features were: GLRLM_RLN_LLL_4gl, GLRLM_LRHGLE_LLL_16gl, GLRLM_LRHGLE_LLL_32gl, GLSZM_SzVarianc_LHL_256gl, original_shape_LeastAxisLength, original_glrlm_LongRunHighGrayLevelEmphasis, n25, and Age. In the feature name, RLN denotes run_length_non_uniformity; SzVarianc is Small Zone variance, n25 is the image intensity 25 percentile from the image mask region, and Age is the age at diagnosis.

In addition, if we include cancer grade as a predictor in the model, the results for classifying cancer histology type were shown in Figure S4 (8 selected features including cancer grade). Figure S4 A shows the selected bar plot of the features. In Figure S4, B, C, and D shows the ROC curve, precision-recall curve, and confusion matrix, respectively. The AUC was 0.97 (with 95% CI was [0.94,1.0]), and the F1 was 0.86 in spite of the unbalanced dataset.

Comparing Figure S4 with Figure 5 A, B, and C, the AUC and F1 values are larger in Figure S4, suggesting cancer grade play an important role for the cancer histological type prediction.

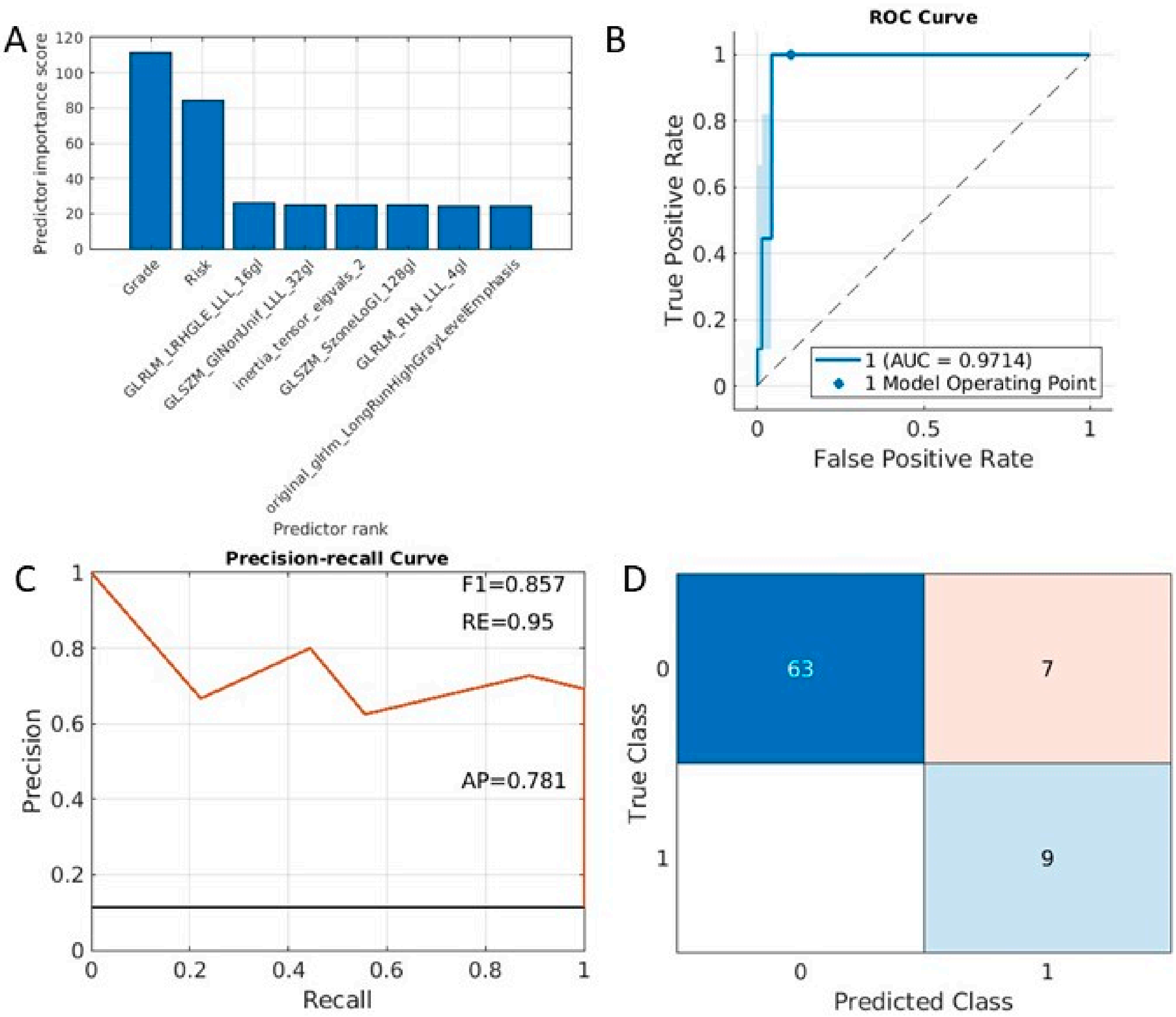

Figure S4. Histological type prediction results including grade as a predictor. Cancer histological type prediction using clinical (including cancer grade cancer stage and clinical risk) and radiological features. A, features included in the model. B, AUC of ROC for the classification. C, precision-recall curve, D, confusion matrix.

Features for LVSI classification

Because there were missing values in some of the target variables, the total number of cases included in each classification study was smaller or equal to 413 cases using training dataset. For the LVSI study, there were only 389 training subjects (of which 141 LVSI positive cases) and 71 (of which 27 LVSI positive cases) testing subjects included in the study (Table 1)

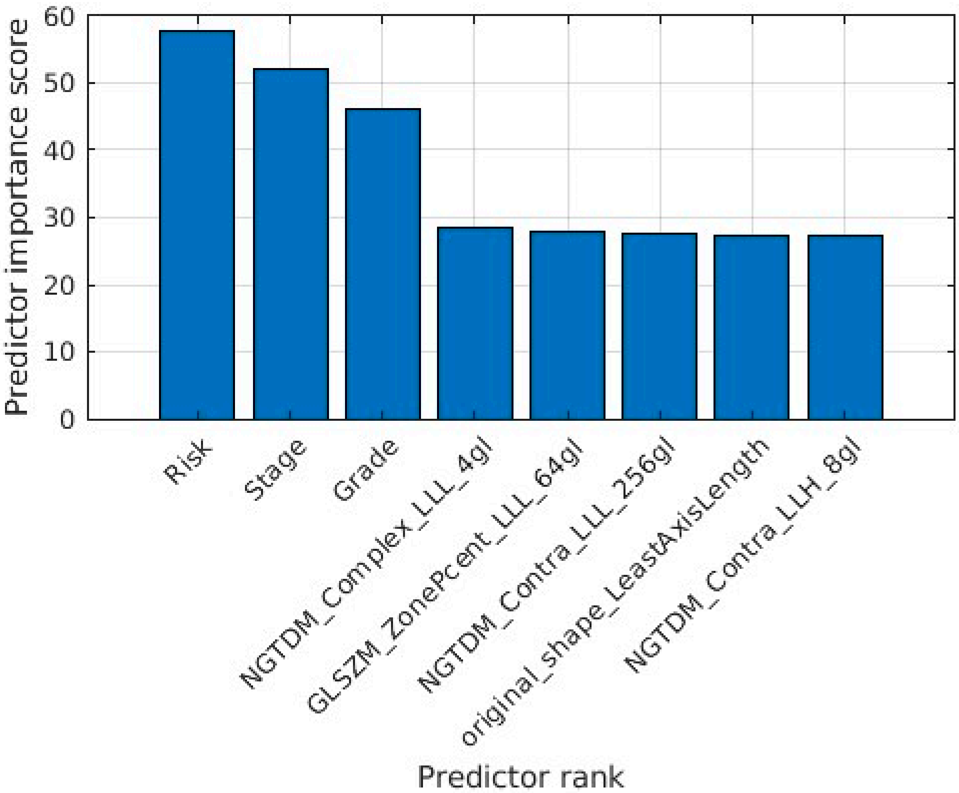

Figure S5. Feature importance bar plot for LVSI prediction. Feature importance score for the most important 8 features for LVSI classification. The Y axis is the importance score measured by the -log(p) value. The X axis is the name of the feature.

The univariate method with individual chi-square tests was used to determine the 8 most important features for the fitcauto method, these features were: cancer Risk, cancer Stage, cancer Grade, NGTDM_Complex_LLL_4gl, GLSZM_ZonePcent_LLL_64gl, NGTDM_Contra_LLL_256gl, original_shape_LeastAxisLength, and NGTDM_Contra_LLH_8gl. In the feature name, ZonePcent is Zone Percentage.

Using the LASSO method, the following features were selected: NGTDM_Complex_LLL_4gl, GLCM_invVar_LLL_8gl, GLCM_difEnt_LLH_8gl, FD_min_LLH_128gl, GLSZM_ZoneLoGl_LHL_8gl, GLCM_sumAvg_LHL_8gl, FD_min_HHH_256gl, and original_shape_LeastAxisLength. In the feature name, ZoneLoGl denotes Low Gray level Zone Emphasis.
